# Supplementary figures and images for: Structural and Functional Analyses of Hub MicroRNAs in An Integrated Gene Regulatory Network of Arabidopsis
Source: Genomics Proteomics Bioinformatics. 2021 Mar 2;20(4):747–64. doi: 10.1016/j.gpb.2020.02.004 (PMC9880815; doi:10.1016/j.gpb.2020.02.004)

**A**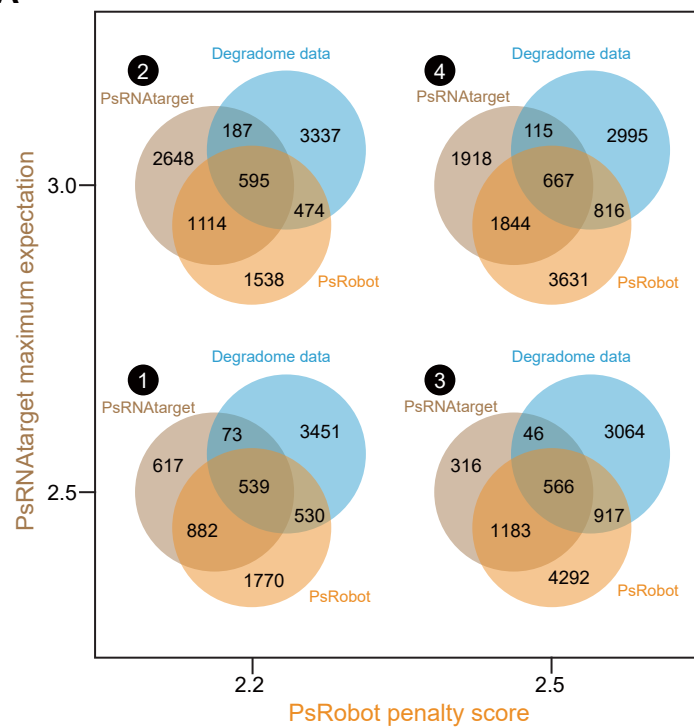**B**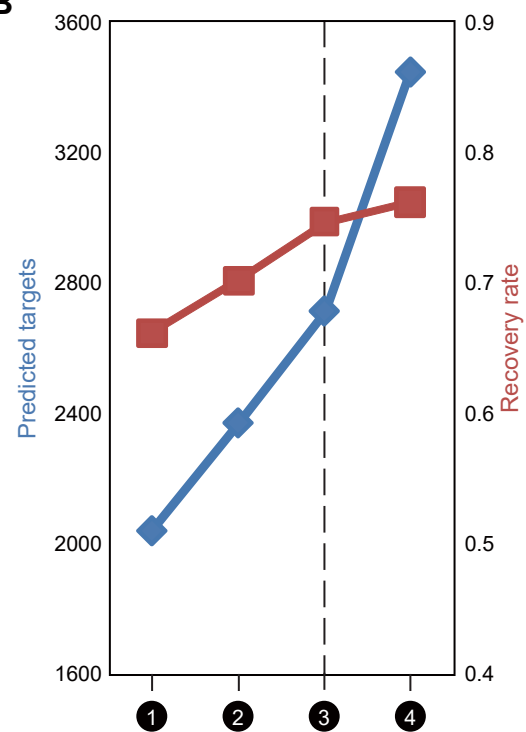**C**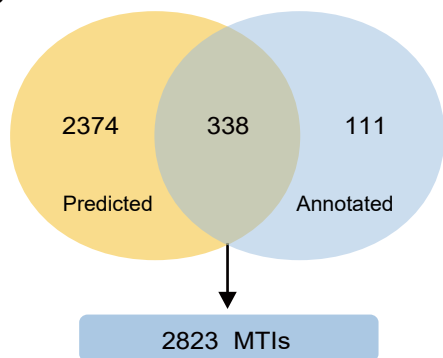**D**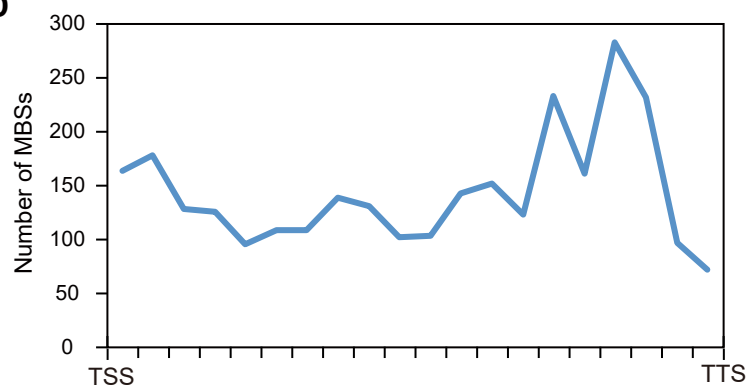

Supplement: Supplementary Figure S1 — Compilation of MTIs through computational prediction and degradome sequencing analysis A. Generation of four datasets of putative MTIs based on computational predictions. Outputs from psRNATarget (maximum expectation score set as 2.5 or 3.0) and psRobot (penalty score set as 2.2 or 2.5) were compared against degradome sequencing data. Four subsequent datasets were generated with each, including putative targets predicted by both programs or by either program but compatible with degradome data. B. Selection of the optimal dataset for representation of MTIs. The four datasets were tested against a benchmark of 449 validated targets to calculate recovery rate and total number of predictions. The dataset that combined output from psRNATarget (2.5), psRobot (2.5), and the degradome data was considered optimal. C. Pie graph showing the final set of 2823 MTIs that includes 2712 predictions and 111 canonical targets not recovered by prediction. D. Distribution of the MBS along the target genes. The 2008 target genes from the 2823 MTI were aligned from head to tail and divided into 20 intervals. The number of MBSs in each interval was calculated and plotted. [file mmc1.pdf]

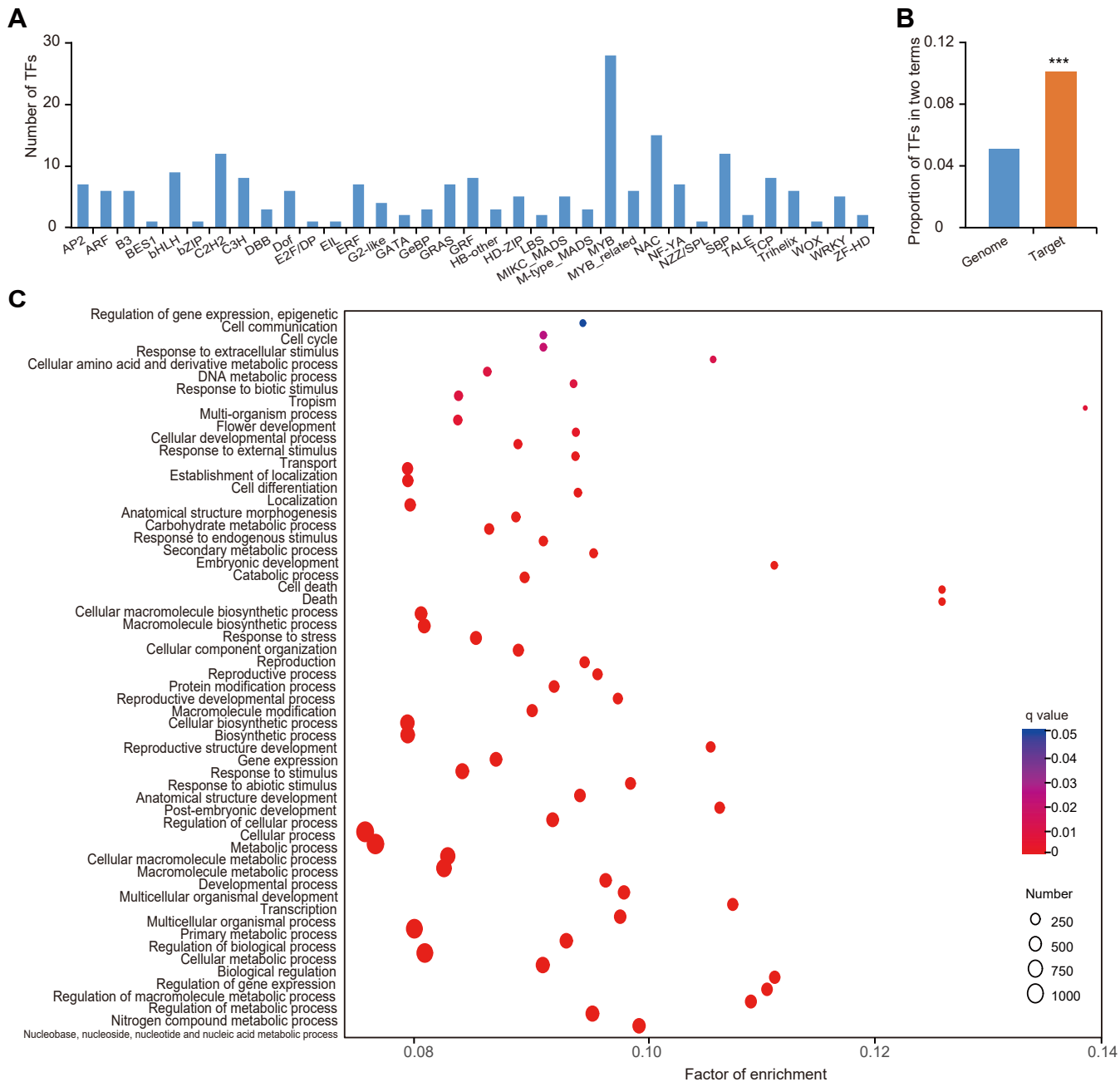

Supplement: Supplementary Figure S2 — TFs are enriched in miRNA target genes A. TF families targeted by miRNAs. A total of 35 TF families in Arabidopsis were found to include at least one member targeted by MTIs. B. Enrichment of TFs in miRNA targets. The proportion of TFs targeted by miRNAs was significantly higher than that at the genome level. ***, P < 0.001 by chi-square test. C. Bubble graph showing GO term analysis of miRNA target genes. The horizontal coordinate represents the factor of enrichment, which is the relative frequency for a given term in the query set against that in the genome. The vertical coordinate represents enriched GO terms in the miRNA targets, with a false-discovery rate (FDR) cutoff of 0.05. The size of the ovals represents the number of genes associated with a given term. The colors of the ovals represent the Q values that indicate the minimum FDR at which significant enrichment of a GO term was determined. [file mmc2.pdf]

**A**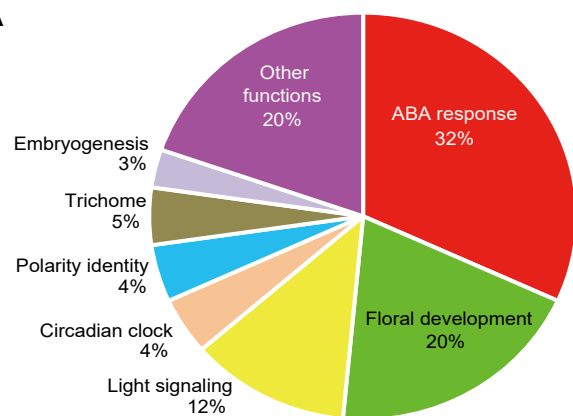**C**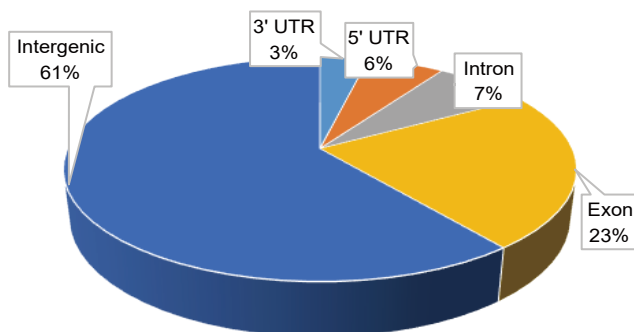**B**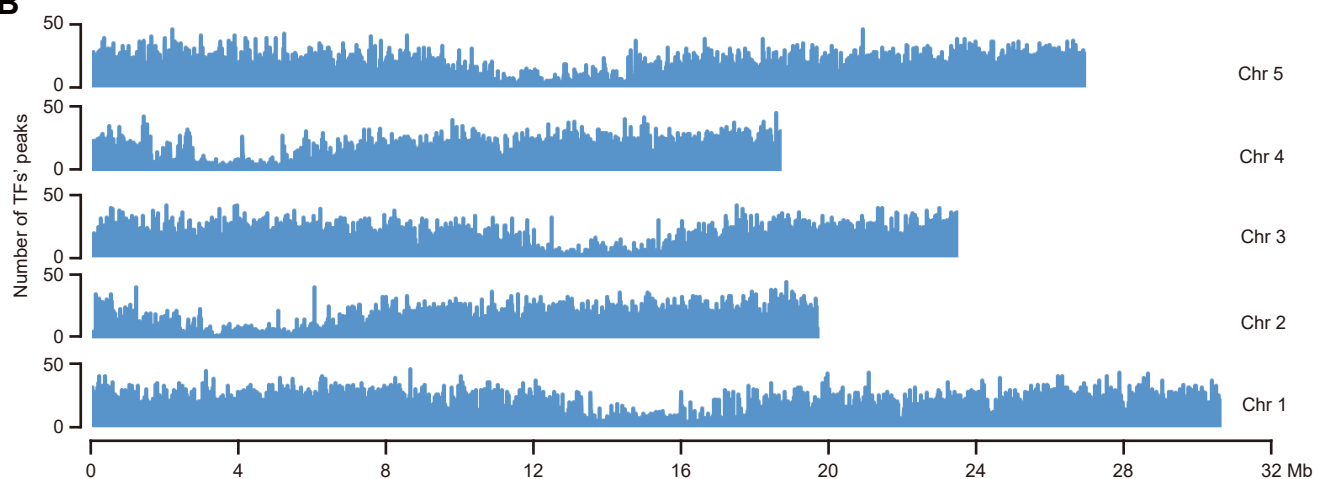

Supplement: Supplementary Figure S3 — Distribution patterns of TF-binding peaks within the genome A. The 66 core TFs with qualified global ChIP data were divided into eight non-overlapping groups based on their annotated functions. B. Chromosome-level distribution of TF binding peaks. A total of 339,875 binding peaks were identified for the 66 TFs after quality control and uniform processing. Each of the five chromosomes was divided into 1000 bp bins, and a sliding window was used to calculate the number of binding peaks. The average number of binding peaks in each bin was plotted against the chromosomal coordinates. C. Localization of the binding peaks in relation to annotated genome components. Percentages of the binding peaks located in 3' UTR, 5' UTR, intronic, exonic, and intergenic regions are shown. [file mmc3.pdf]

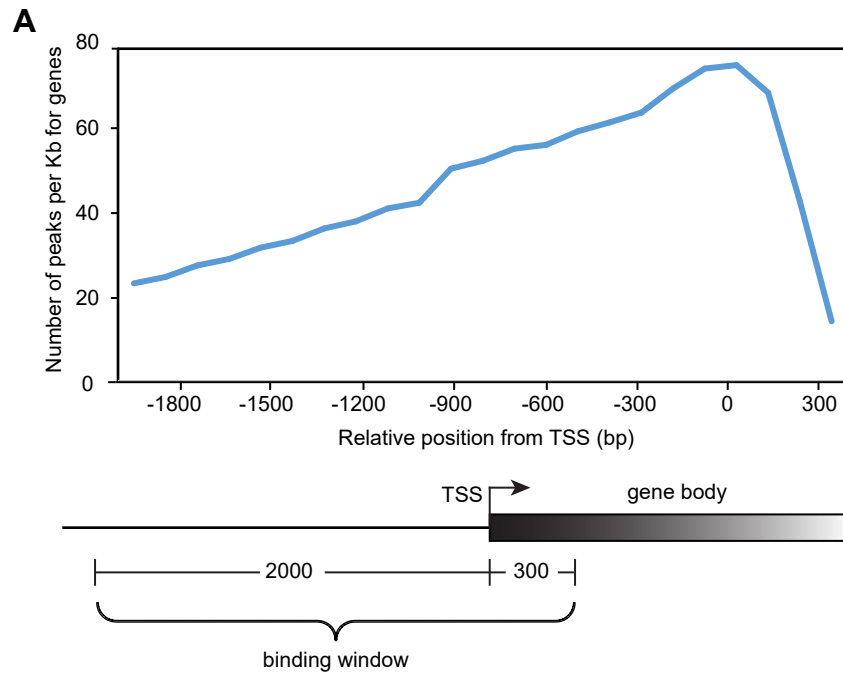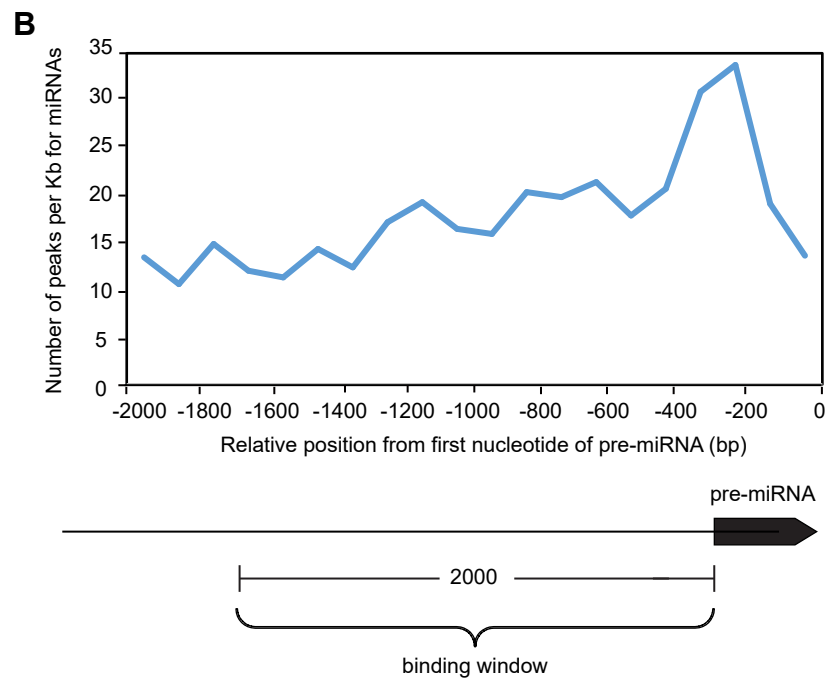

Supplement: Supplementary Figure S4 — Mapping TTIs and TMIs using the TF binding peaks A. Gene-level distribution of TF binding peaks for protein-coding loci. A sliding window analysis showed that binding peaks concentrate at the TSS and extend 2000 bp upstream and 300 bp downstream of the TSS. This region was defined as a binding window. If the midpoint of any of the 339,875 binding peaks was identified in the binding windows, a putative TTI was declared between the corresponding TF and the given protein-coding gene. B. For miRNA loci, the binding peaks were found to concentrate at the 200 bp region upstream of the first nucleotide of the annotated pre-miRNAs. The 2000 bp region upstream of the first nucleotide of the pre-miRNA was defined as the binding window for establishing TMIs for the miRNA loci. [file mmc4.pdf]

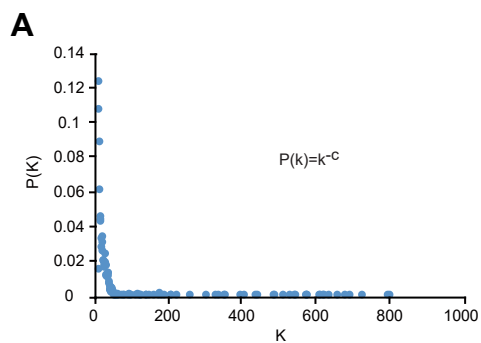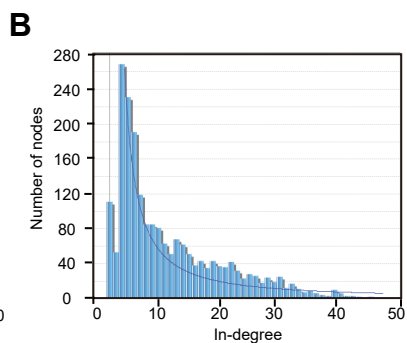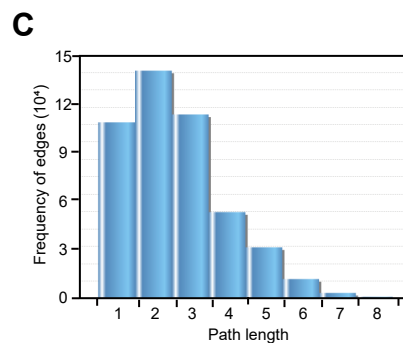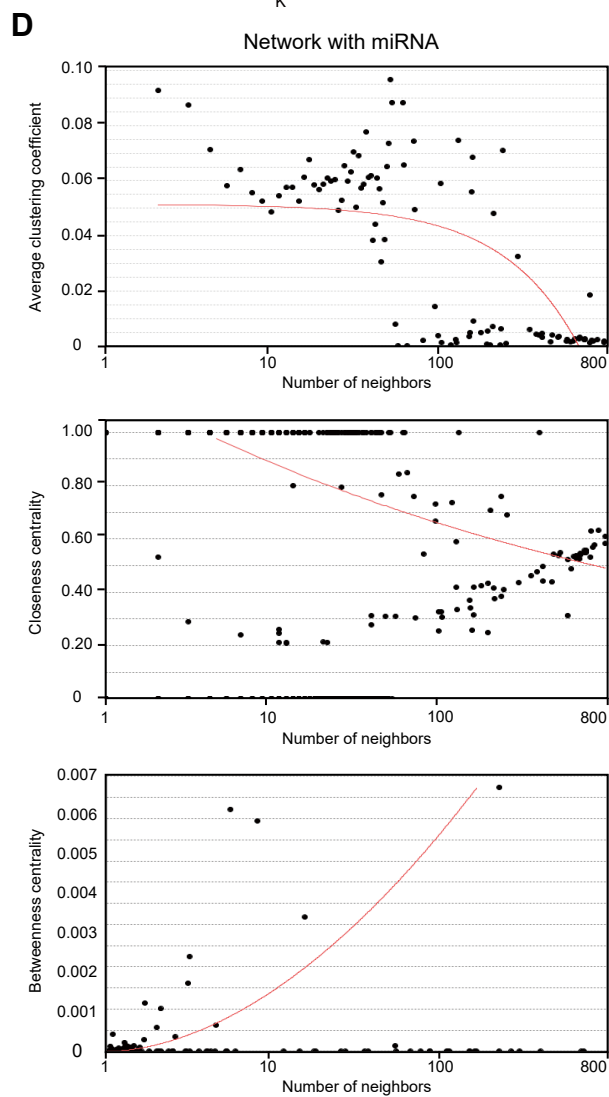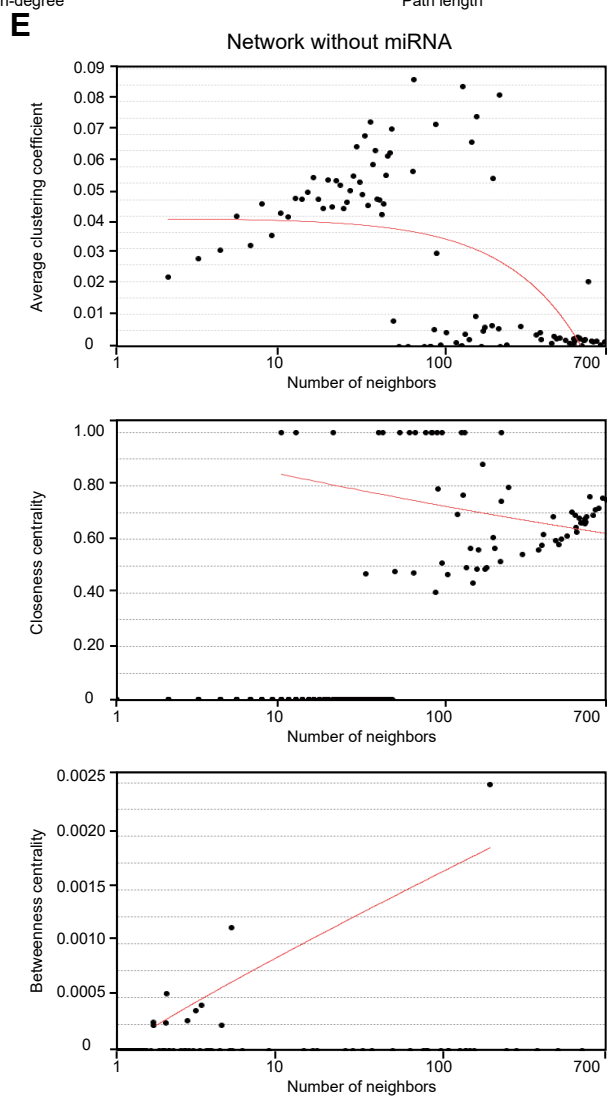

Supplement: Supplementary Figure S5 — Topological analysis of the reconstructed miRNA network A. Distribution of node degree in the network. The degree of all nodes was calculated and the frequency distribution plotted, which followed a power-law. B. Distribution of the node in-degree. Only the in-degree of all nodes was calculated and plotted. C. Histogram showing frequency distribution of the path length. All connected nodes in the network were used to extract the paths, and their lengths (steps) were calculated. D. Node properties in the network. Using the NetworkAnalyzer function in Cytoscape, clustering coefficient, closeness centrality, and betweenness centrality for all nodes in the network were determined and their respective distributions plotted. E. Distribution of clustering coefficient, closeness centrality, and betweenness centrality after all miRNA nodes were removed. [file mmc5.pdf]

**A**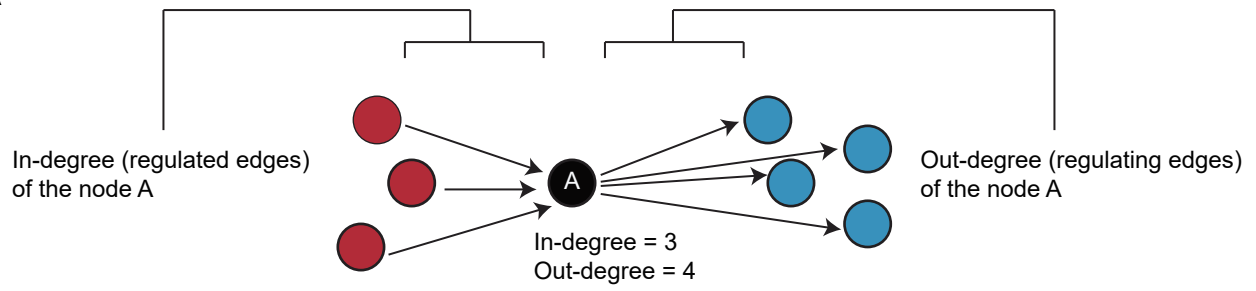**B**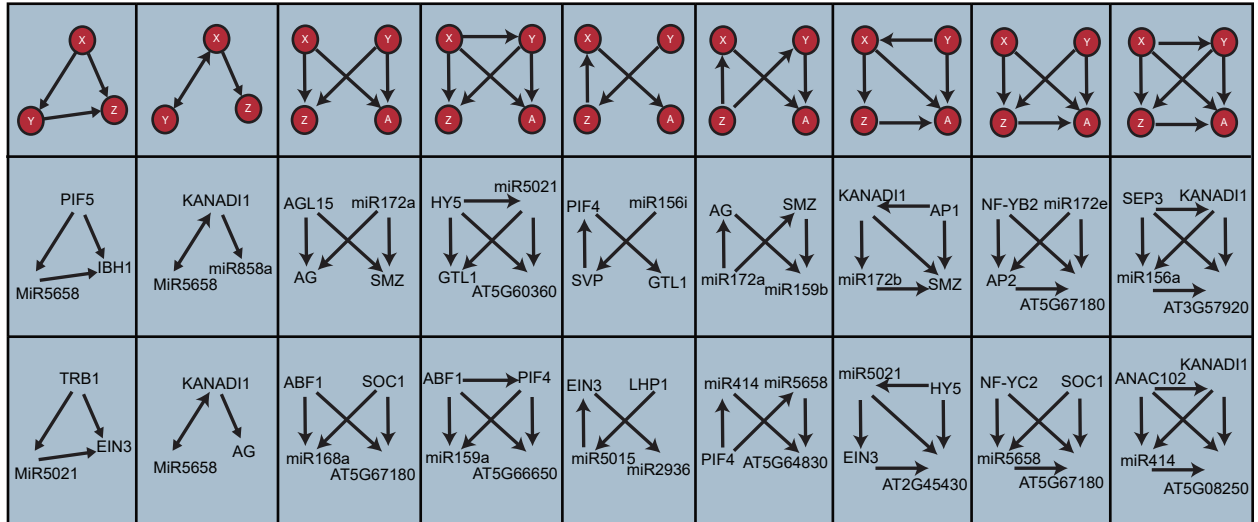

Supplement: Supplementary Figure S6 — Enrichment of miRNA-containing network motifs in the reconstructed miRNA network A. Diagram showing the in-degree (regulated edges) and out-degree (regulating edges) of a given node. B. Enriched miRNA-containing motifs (Z score > 2) in the reconstructed network. Graphic representation of the motifs and a specific example for each enrich motif are shown. [file mmc6.pdf]

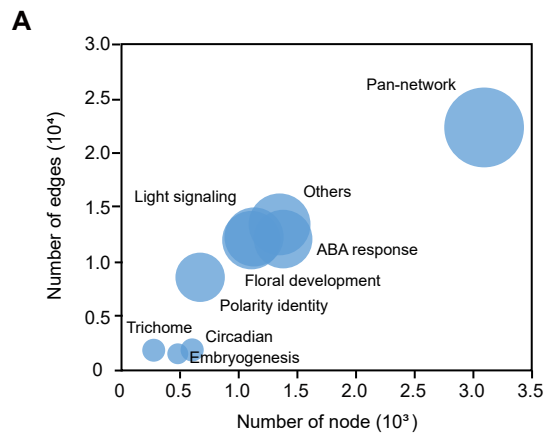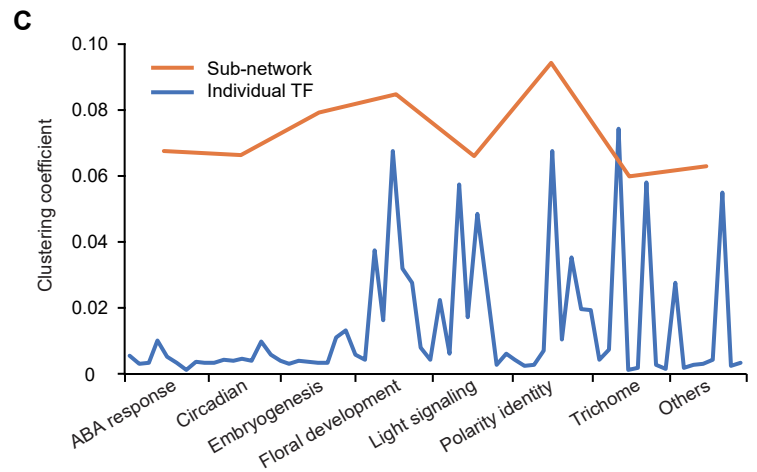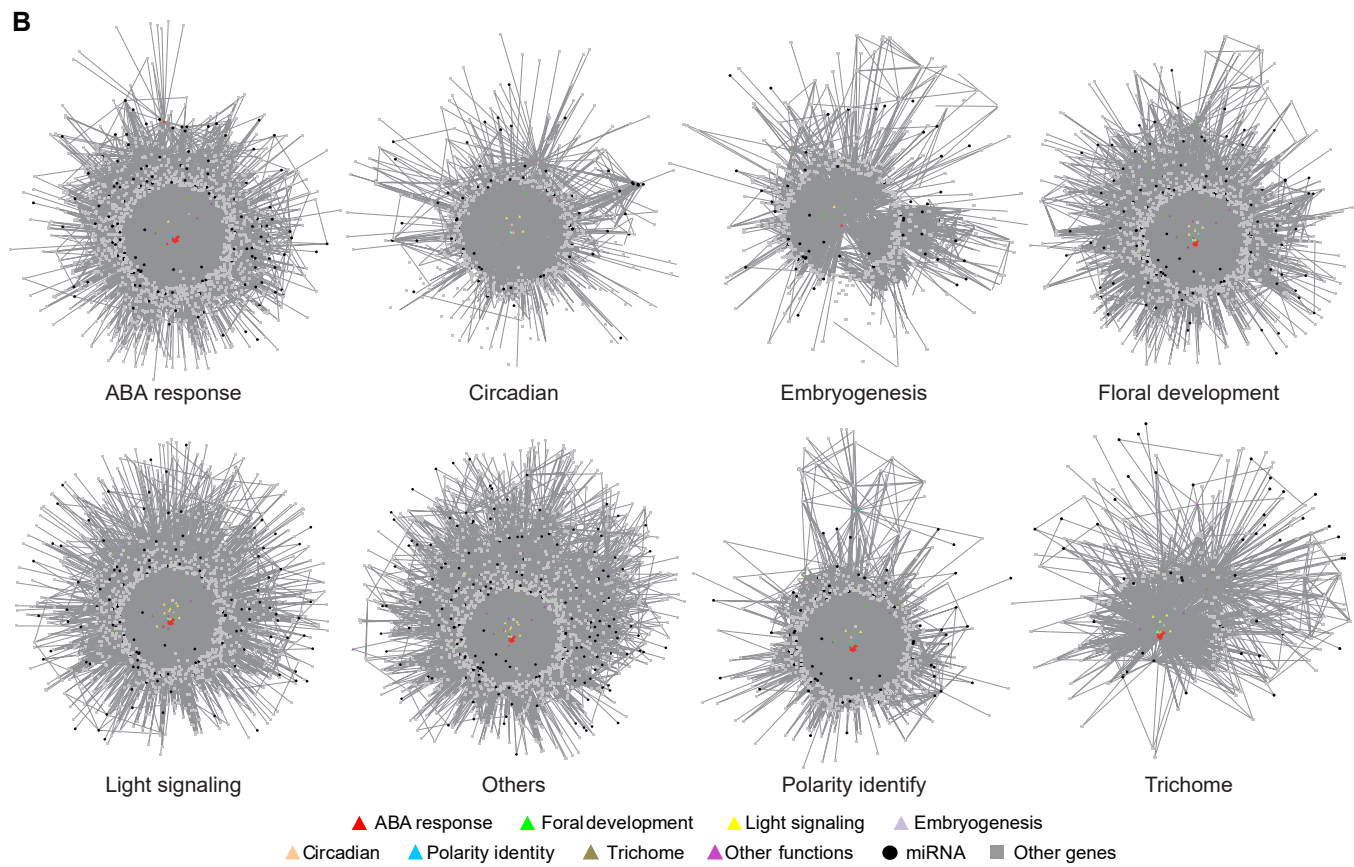

Supplement: Supplementary Figure S7 — The miRNA network contains eight modular TF-defined sub-networks A. Bubble chart displaying the number of nodes and edges of the eight TF-defined sub-networks together with the pan-network. The size of the disks represents the number of edges. B. Visualization of the eight sub-networks reconstructed from the pan-network based on the distinctive functions of the core TFs. The core TFs are shown as colored triangles, miRNAs as black circles, and other genes as gray squares. All edges are shown as gray lines. C. Node clustering coefficient at the sub-network and individual TF levels. Values are the average clustering coefficient for the eight sub-networks and the average of all nodes connected to a TF through TTIs and TMIs. [file mmc7.pdf]

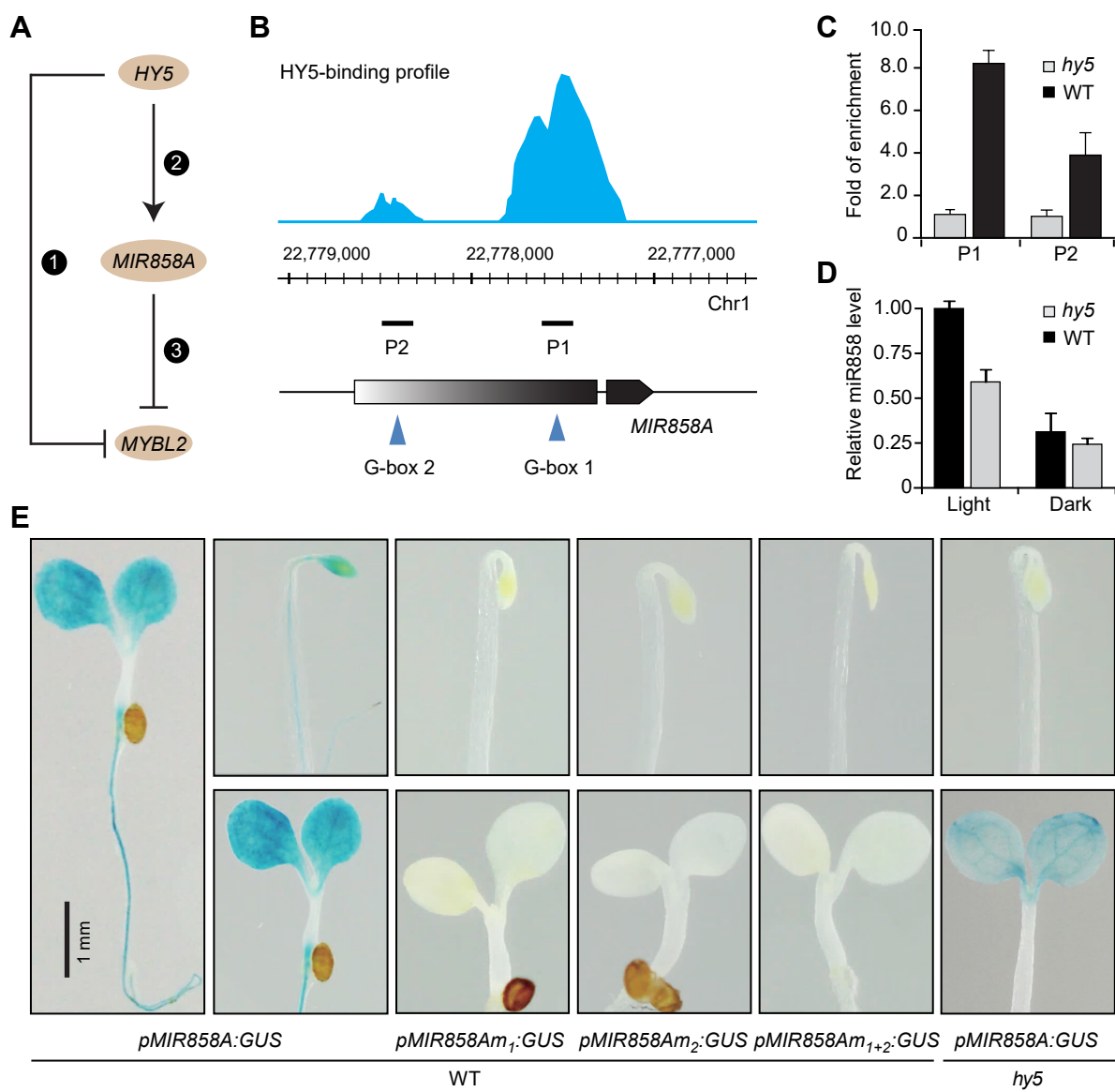

Supplement: Supplementary Figure S8 — Validation of HY5-MIR858A-MYBL2 as a coherent FFL A. Structure of the HY5-MIR858A-MYBL2 FFL, which consists of three edges forming a direct path and an indirect path. All three regulatory interactions were validated by multiple experimental approaches, which collectively manifested the FFL as a coherent type. B. HY5 occupancy at the MIR858A locus based on global ChIP data mapped onto genome coordinates. The pre-miR858a and promoter region are depicted as a black arrow and a horizontal bar, respectively. The triangles mark the two G-boxes in the promoter region. P1 and P2 indicate the positions of the two amplicons for ChIP-qPCR analysis. C. Confirmation of HY5 binding to the MIR858A promoter by ChIP-qPCR analysis. ChIP was performed in the wild type and hy5 seedlings using an anti-HY5 antibody. The precipitated DNA was analyzed by qPCR, with the values normalized to those of the IgG-treated samples. D. Analysis of mature miR858 transcript levels under light and dark conditions. Levels of mature miR858 determined by qRT-PCR were normalized, and the value for light-grown wild type seedlings was set to 1. Data for ChIP-qPCR and qRT-PCR represent means ± SD (n = 3). E. GUS activity driven by various forms of the MIR858A promoter expressed in the wild type or hy5 background. Seedlings grown under dark (top) and light (bottom) conditions were stained for GUS activity and visualized. Bar, 1 mm. [file mmc8.pdf]

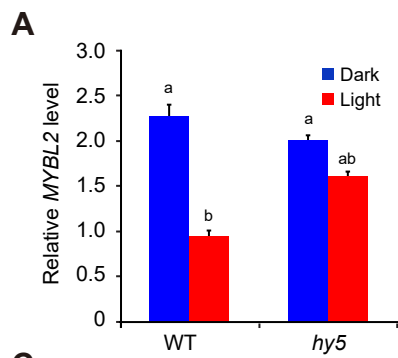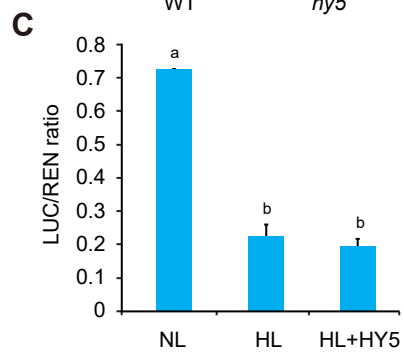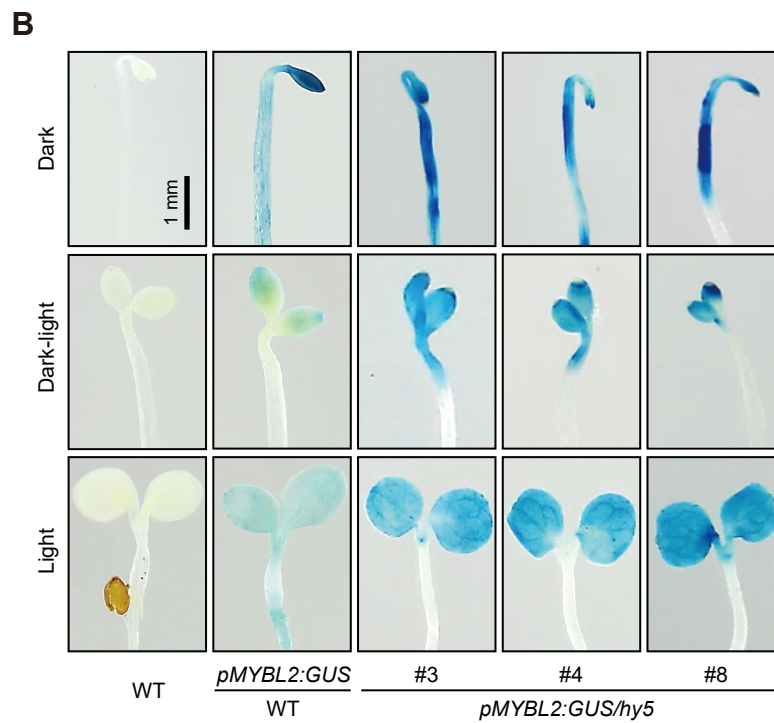

Supplement: Supplementary Figure S9 — HY5 partially mediates suppression of MYBL2 transcription by light A. qRT-PCR analysis of MYBL2 transcript levels in wild type and hy5 seedlings grown in the dark and light. MYBL2 levels were normalized, and the value for wild type in light was set to 1. Data are means ± SD (n = 3). B. Analysis of HY5 dependent MYBL2 promoter activity. Transgenic seedlings expressing pMYBL2:GUS in the wild type or hy5 background were subjected to different light treatments and assayed for GUS activity. Dark-light, transitioning from dark to light for 12 h. Note the difference in seedling morphology influenced by the light treatments. Bar, 1 mm. C. Suppression of MYBL2 promoter activity by HY5. The REN/LUC dual luciferase reporter construct, in which LUC is driven by the native MYBL2 promoter, was employed. Tobacco protoplasts were transformed with this reporter alone or together with 35S:HA-HY5 and incubated overnight in either the dark or light. The ratio of the LUC/REN chemiluminescence values was then determined. Data are means ± SD (n = 2). [file mmc9.pdf]

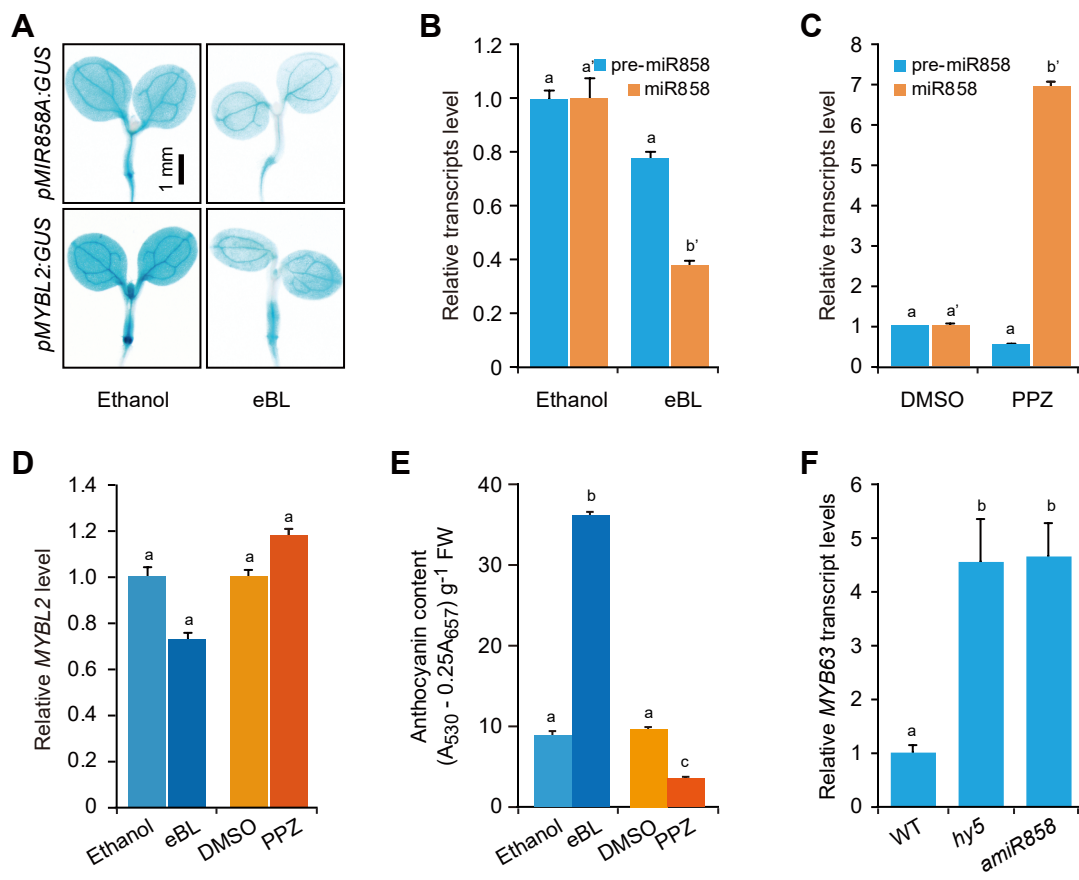

Supplement: Supplementary Figure S10 — Molecular analyses of intertwined FFLs centered on MIR858A A. Promoter activity of MIR858A and MYBL2 in response to eBL treatments. Seedlings expressing pMIR858A:GUS (top) or pMYBL2:GUS (bottom) were treated with ethanol or eBL and stained for GUS activity. Bar, 1 mm. B. and C. Analysis of pre-miR858a and mature miR858 transcript levels in seedlings after eBL (B) and PPZ treatment (C) in comparison to the respective mock treatment by qRT-PCR analysis. D. Change in MYBL2 transcript level in seedlings following eBL and PPZ treatments. E. Exogenous eBL and PPZ treatment drastically increases and decreases anthocyanin levels, respectively, in Arabidopsis seedlings. Samples labeled with different letters denote groups with significant differences (one-way ANOVA test, P < 0.05). All eBL and PPZ treatments were 0.1 µM. F. Comparison of MYB63 transcript levels in the inflorescence stem of the hy5 and amiR858 mutants to that of the wild type. All qRT-PCR data are means ± SD (n = 3). [file mmc10.pdf]

**A**

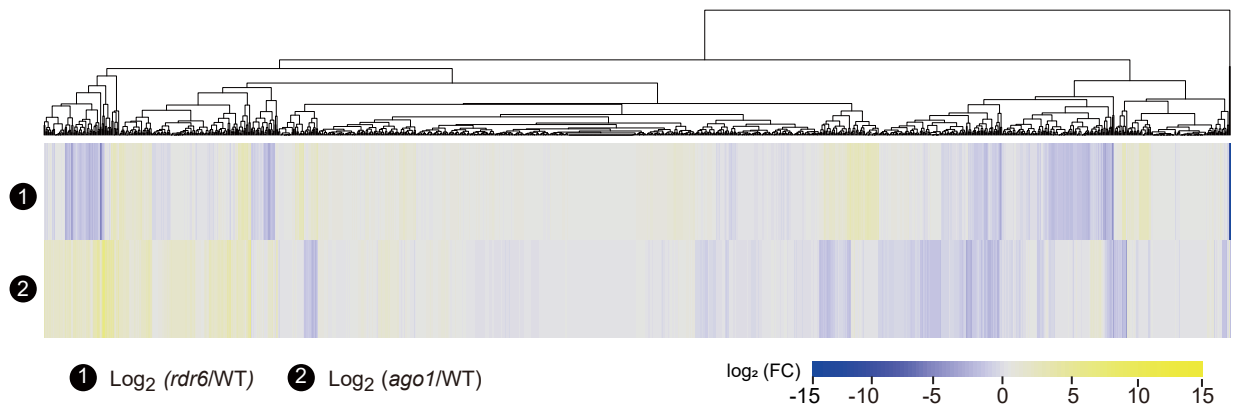

**B**

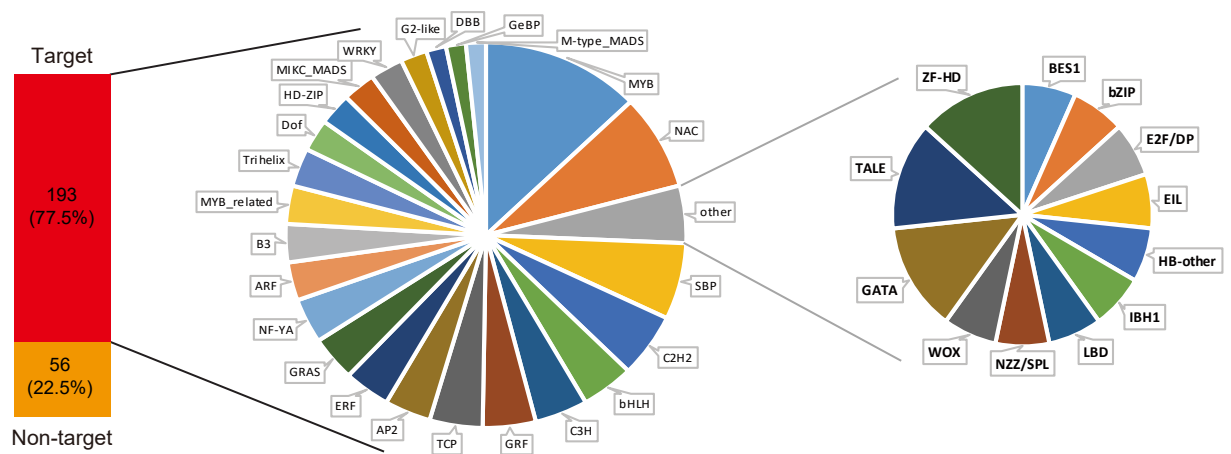

Supplement: Supplementary Figure S11 — Analysis of TFs related to the core network A. Heatmap displaying the relative expression levels of the 1717 annotated TFs in Arabidopsis mutants with defective small RNA pathways. Log2-transformed expression levels in rdr6 and ago1 mutants compared to that of the wild type are shown. B. Analysis of the 249 TFs in the miRNA-TF core network. The proportions of TFs that were miRNA targets and non-targets are shown as stacked bars on the left. The pie graphs in the middle and on the right show family information for the miRNA-targeted TFs. [file mmc11.pdf]
